# A physiology-based approach to a patient with hyperkalemic renal tubular acidosis

**DOI:** 10.1590/2175-8239-JBN-3821

**Published:** 2018-07-23

**Authors:** Juliana Menegussi, Luiza Sarmento Tatagiba, Júlia Guasti P. Vianna, Antonio Carlos Seguro, Weverton Machado Luchi

**Affiliations:** 1 Universidade Federal do Espírito Santo, Vitória, ES, Brasil.; 2 Universidade de São Paulo, Faculdade de Medicina, Departamento de Nefrologia, Laboratório de Pesquisa Médica - LIM12, São Paulo, SP, Brasil.; 3 Universidade Federal do Espírito Santo, Departamento de Clínica Médica, Divisão de Nefrologia, Vitória, ES, Brasil.

**Keywords:** Hyperkalemia, Calcineurin, Hypoaldosteronism, Acidosis, Renal Tubular, Magnesium, Hipercalemia, Calcineurina, Hipoaldosteronismo, Acidose Tubular Renal, Magnésio

## Abstract

Hyperkalemic renal tubular acidosis is a non-anion gap metabolic acidosis that invariably indicates an abnormality in potassium, ammonium, and hydrogen ion secretion. In clinical practice, it is usually attributed to real or apparent hypoaldosteronism caused by diseases or drug toxicity. We describe a 54-year-old liver transplant patient that was admitted with flaccid muscle weakness associated with plasma potassium level of 9.25 mEq/L. Additional investigation revealed type 4 renal tubular acidosis and marked hypomagnesemia with high fractional excretion of magnesium. Relevant past medical history included a recent diagnosis of Paracoccidioidomycosis, a systemic fungal infection that is endemic in some parts of South America, and his outpatient medications contained trimethoprim-sulfamethoxazole, tacrolimus, and propranolol. In the present acid-base and electrolyte case study, we discuss a clinical approach for the diagnosis of hyperkalemic renal tubular acidosis and review the pathophysiology of this disorder.

## RESUMO

A acidose tubular renal hipercalêmica é uma acidose metabólica de ânion gap normal que invariavelmente indica anormalidade na secreção de íons potássio, amônio e hidrogênio. Na prática clínica, está geralmente atribuída a um estado de hipoaldosteronismo real ou aparente, causado por doenças ou toxicidade por drogas. Descrevemos um paciente de 54 anos, transplantado hepático, que foi admitido com fraqueza muscular associada à hipercalemia, potássio plasmático de 9,25 mEq/L. A investigação adicional revelou acidose tubular renal tipo 4 e importante hipomagnesemia com elevada fração de excreção de magnésio. A história patológica pregressa incluía um diagnóstico recente de Paracoccidioidomicose - uma infecção sistêmica fúngica endêmica que ocorre em algumas partes da América do Sul -, e as medicações de uso habitual continham sulfametoxazol-trimetoprim, tacrolimus e propranolol. No presente relato de caso, discutiremos uma abordagem clínico-laboratorial para o diagnóstico da acidose tubular renal hipercalêmica, assim como da hipomagnesemia, revisando a fisiopatologia desses transtornos.

## INTRODUCTION

Renal tubular acidosis (RTA) is a group of syndromes arising from different transport defects in bicarbonate reabsorption or hydrogen excretion. Despite the presence of renal tubular dysfunction, the glomerular filtration rate (GFR) is relatively preserved in RTA. The condition is characterized by non-anion gap or hyperchloremic metabolic acidosis associated with positive urinary anion gap (AG) and can be accompanied by low, normal or high serum potassium concentration. Hyperkalemic RTA, also called type 4 RTA, invariably implies an abnormal potassium, ammonium, and proton secretion. It is linked to conditions affecting lumen-negative voltage gradient generated by sodium reabsorption in the collecting duct (CD) and the ammoniagenesis within proximal tubular cells, usually attributed to real or apparent hypoaldosteronism. With the following case study, we describe our approach to a patient with severe hyperkalemic RTA and hypomagnesemia, highlighting pathophysiologic mechanisms and important key points to the diagnosis.

## CASE REPORT

### CLINICAL HISTORY AND INITIAL LABORATORY DATA

A 54-year-old man, who underwent a liver transplant two years ago as a treatment for end-stage liver disease caused by alcoholic cirrhosis, was admitted because of a 4-week progressive muscle weakness involving the lower and upper extremities. He was unable to walk alone at presentation and physical examination revealed flaccid weakness of proximal muscles (2/5 strength grade) without hypotrophy or sensory deficit. He was hydrated, had regular heart rhythm (60 bpm), blood pressure of 120/80 mmHg, and unremarkable pulmonary and abdominal examinations. The man had no previous medical history of hypertension, diabetes mellitus or kidney disease. He also described that six months earlier, he started treatment with trimethoprim-sulfamethoxazole due to the appearance of diffuse nodules in the skin and subcutaneous, the biopsy of which was consistent with paracoccidioidomycosis (PCM). Other outpatient medications were propranolol for prevention of esophageal variceal bleeding and tacrolimus for prophylaxis against graft rejection.

**Figure 1:**
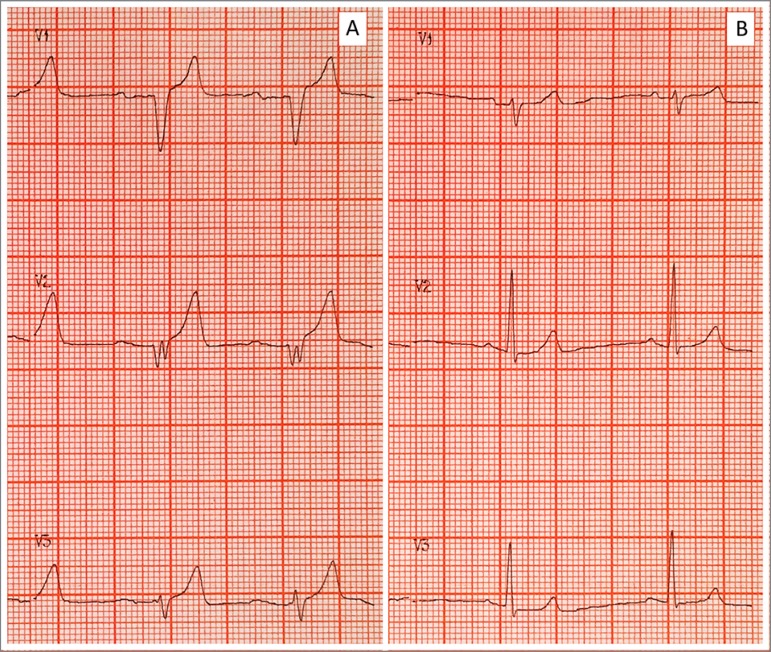
(A) Pretreatment electrocardiogram with peaked T-waves, flattening of the P-wave, prolonged PR interval, and widening of the QRS complex. (B) Post-treatment electrocardiogram with normalization of T-waves, PR, and QRS intervals.

Initial laboratory tests (Table 1) showed severe hyperkalemia (9.25 mEq/L) and the electrocardiogram revealed "peaked" T waves, widened and flattened P waves, prolonged PR interval, and widened QRS complex, as illustrated in Figure 1A. Immediate stabilization of the myocardial cell membrane with iv injection of 10 mL of 10% calcium gluconate over two minutes and rapid shifting of potassium to the intracellular space by iv injection of insulin with glucose (10 units of regular insulin plus 100 mL of 50% glucose in 30 minutes), 8.4% sodium bicarbonate (150 mEq IV in 30 minutes), and beta-agonists inhalation (fenoterol 20 drops = 5 mg) were the initial priorities. After these interventions, the electrocardiogram normalized (Figure 1B). Volume expansion with 0.9% saline solution (2 L in 2 hours) followed by iv injection of 40 mg furosemide generated a high urinary volume that contributed for body potassium elimination. Due to the persistence of severe acidosis, another infusion with 100 mEq of bicarbonate was performed. Calcium polystyrene sulfonate, a chelating agent, was subsequently given (30 g orally three times a day) because of its delayed action.

**Table 1:** Laboratory Parameters

Blood	On Admission	Day 2 --> Day 5	Reference Range
Creatinine (mg/dL)	1.8	1.5 --> 0.8	0.7-1.2
Urea (mg/dL)	115	84 --> 32	10-50
Calcium (mg/dL)	9.79		8.8-10.5
Chloride (mEq/L)	113		98-106
Magnesium (mg/dL)	1.4	1.2 --> 1.6	1.8-2.4
Potassium (mEq/L	9.25	5.8 --> 4	3.5-5.1
Sodium (mEq/L)	137		135-145
Glycated hemoglobin (%)	5.5		< 6
Arterial Blood Gas			
pH	7.247		7.35-7.45
pCO_2_ (mmHg)	23.7		35-40
HCO_3_ (mEq/L)	12.7	19 --> 21	22-26
Anion Gap (mEq/L)	11.3		10±2
Renin Activity (ng/mL/h)	9.2		0.2-3.3
Aldosterone (ng/dL)	13.8		2.5-39.2
Basal Cortisol (µg/dL)		7.8	6.2-19.4
ACTH (pg/dL)		10	< 46
Tacrolimus level (ng/dL)	27.8		5-7
Urine (spot)			
pH	5.0		4.5-8
Sodium (mEq/L)	117		20-110
Chloride (mEq/L)	130		55-125
Potassium (mEq/L)	31		12-62
TTKG	2.3		~ 4-6
FE Mg (%)	9		2-4
Anion Gap (mEq/L)	+ 18		negative

### ADDITIONAL INVESTIGATIONS

Once hyperkalemia was identified and therapeutic interventions initiated, a urine sample was promptly collected. It is important to emphasize that when an electrolytic disturbance is detected, a urine sample must be immediately collected, since therapeutic interventions may alter pH and electrolyte concentrations in the urine, possibly distorting correct interpretations and diagnosis. Urine tests in the emergency department have short turnaround time, usually within one hour, and can be helpful to guide the correct diagnosis and treatment.

As depicted in Table 1, arterial blood gas revealed marked metabolic acidosis with normal serum anion-gap (plasma [Na^+^] - [HCO3^-^] - [Cl^-^]), and an isolated urine sample showed apparent noraml urinary acidification (urine pH: 5.0). Urinary AG (urine [Na^+^] + [K^+^] - [Cl^-^]) was +18 and calculated transtubular potassium gradient was 2.3 (TTKG = [K^+^_urine_^*^ Osm_plasma_] / [K^+^_plasma_^*^ Osm_urine_]). Urine osmolality can be estimated using the following formula: Osm_urine_ = (2 ^*^ [Na^+^_mEq/L_ + K^+^_mEq/L_]) + (Glucose_mg/dL_/18) + (Urea_mg/dL_/6). Fractional excretion of magnesium was 9%, calculated by FE_Mg%_ = 100^*^ [Mg^+2^_urine_ x Cr_plasma_] / [0.7 ^*^ Mg^+2^_plasma_ x Cr_urine_]. Serum magnesium concentration is multiplied by 0.7 in order to adjust for magnesium filtered by the kidney.

Because of renal hyperkalemia without advanced decreased of GFR, plasma aldosterone and plasma renin activity analysis were required. Serum cortisol, plasma ACTH, and abdominal computed tomography (CT) were indicated since PCM is known to involve the adrenal gland. Drug-induced nephrotoxicity was also evoked as a possible diagnosis and the above-mentioned medications were temporarily suspended and tacrolimus was replaced by mycophenolate.

## DIAGNOSIS

### HYPERKALEMIC RTA AND RENAL MAGNESIUM WASTING

#### CLINICAL FOLLOW-UP

As shown in Table 1, a significant decrease in plasma potassium levels was progressively observed and there was no need for dialysis therapy. Renal function returned to the previous baseline after five days. Further evaluation excluded the hypothesis of adrenal insufficiency associated with PCM despite the identification of an adrenal nodule in the CT. Aldosterone level was inappropriate for hyperkalemia and the main causal factor was very high level of tacrolimus (Table 1). During follow-up, trimethoprim and propranolol were reintroduced, followed by tacrolimus (dose reduction from 4 to 1 mg per day) without new disorders in plasma potassium, bicarbonate or tacrolimus levels. Below, we discuss the differential diagnoses for the case, dissecting the understanding of hyperkalemic RTA and hypomagnesemia.

## DISCUSSION

The presented case illustrates a typical non-anion gap or hyperchloremic metabolic acidosis. Renal or extrarenal causes for this disturbance can be differentiated by urine AG. It indirectly represents the excretion of unmeasured ammonium cation (NH4^+^) that constitutes the most import urinary buffer system to excrete H^+^ during acid overload. If the kidneys do not excrete NH4^+^ properly, the urine AG turns positive, suggesting RTA as the cause of hyperchloremic metabolic acidosis^1^.

Among the RTA types, only type 4 leads to hyperkalemia. Conversely, proximal (type 2) and distal (type 1) occur with normal or low plasma potassium levels. TTKG is a clinically useful tool for estimating the potassium concentration "gradient" between the peritubular capillary and the tubular lumen at the level of cortical CD. A TTKG lower than 8 in the hyperkalemic patient implies that the kidney is not responding appropriately to the prevailing hyperkalemia and that potassium secretion is impaired^2^^,^ 3.

**Figure 2:**
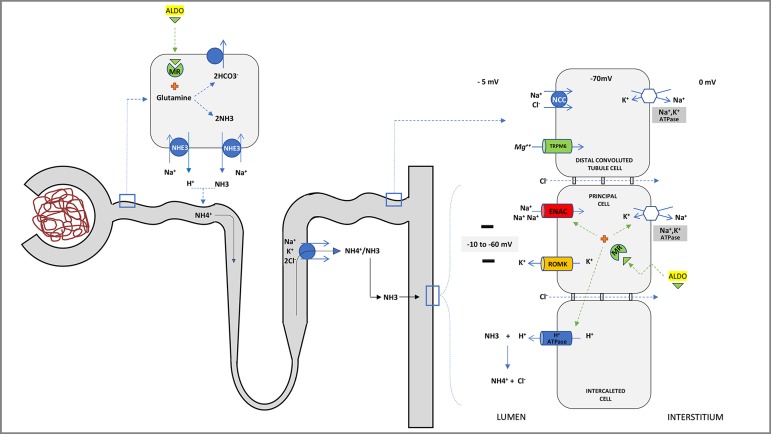
Interaction between potassium and proton excretion and ammoniagenesis. Sodium reabsorption by ENAC transporter in principal cells, driven by Na^+^/K^+^-ATPase, creates a lumen-negative transepithelial voltage that is critical for potassium (by ROMK) and proton (By H-ATPase) excretion in the collecting duct (CD). The excretion of H^+^ also requires the ammonia buffer that prevents a marked drop in urinary pH. Ammonia is produced in the proximal cells from glutamine and reaches tubular fluid as NH_4_^+^. After, it is reabsorbed in the thick ascending limb to the interstitium and then is secreted as NH_3_ into the CD by α-intercalated cells in parallel with the H^+^. Aldosterone (ALDO) is a pivot in these processes, stimulating both sodium reabsorption and ammoniagenesis. Impairment of the ENAC activity and/or Na^+^/K^+^-ATPase transporters, reduction of the amount of sodium delivered in CD, and the reduction in ammonia production are the main mechanisms involved in the pathogenesis of type 4 renal tubular acidosis. MR: mineralocorticoid receptor.

**Figure 3:**
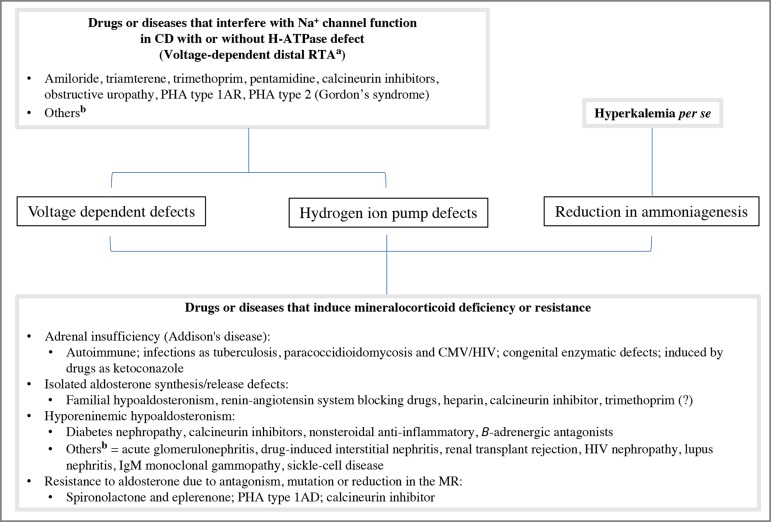
Pathophysiologic classification and etiologies of disorders associated with hyperkalemic hyperchloremic renal tubular acidosis. PHA: pseudohypoaldosteronism; CD: collecting duct; MR: mineralocorticoid receptor. ^a^ = The voltage defect causes a relative "resistance" to aldosterone in the CD, but does not interfere with its action on ammoniagenesis in the proximal cells; ^b^ = Others: Hyperkalemia due to these causes may be related to hyporeninemic hypoaldosteronism and/or a direct defect in voltage gradient generation in CD.

In normal circumstances, the reabsorption of sodium in the CD, driven by aldosterone, generates transepithelial voltage gradient that is lumen-negative, creating a driving force for the secretion of potassium and hydrogen, by principal and α-intercalated cells, respectively (Figure 2). Besides, the proton secretion requires the parallel movement of NH3, and its protonation to NH4^+^, in order to provide sufficient buffering. The ammonia is produced in proximal tubules by glutamine deamidation, reaching the renal medulla through NKCC transporter in the Henle loop. After, it is secreted in urine in the distal nephron. Apart from stimulating Na^+^/K^+^-ATPase, ENaC, and H-ATPase transporters, aldosterone plays a pivotal role in ammoniagenesis^2^^,^ 4 ^,^ 5. Any interference in these pathways may lead to hyperkalemic RTA. The etiologies and pathophysiological mechanisms of hyperkalemic RTA are briefly reviewed in Figure 3.

Urine pH depends on both the concentration of H^+^ and the amount of ammonium buffer. A normal renal response to acidemia includes an ability to produce urine with pH as low as 5.0. Thus, a deficit of proton secretion tends to leave the urine with an inappropriate high pH (>5.5) despite systemic acidosis. However, even with a reduction in H^+^ secretion, the urine pH may remain below 5.5 if an ample reduction the ammonium buffer occurs simultaneously. In this circumstance, the interpretation of adequate urinary acidification will be misleading^6^.

**Figure 4:**
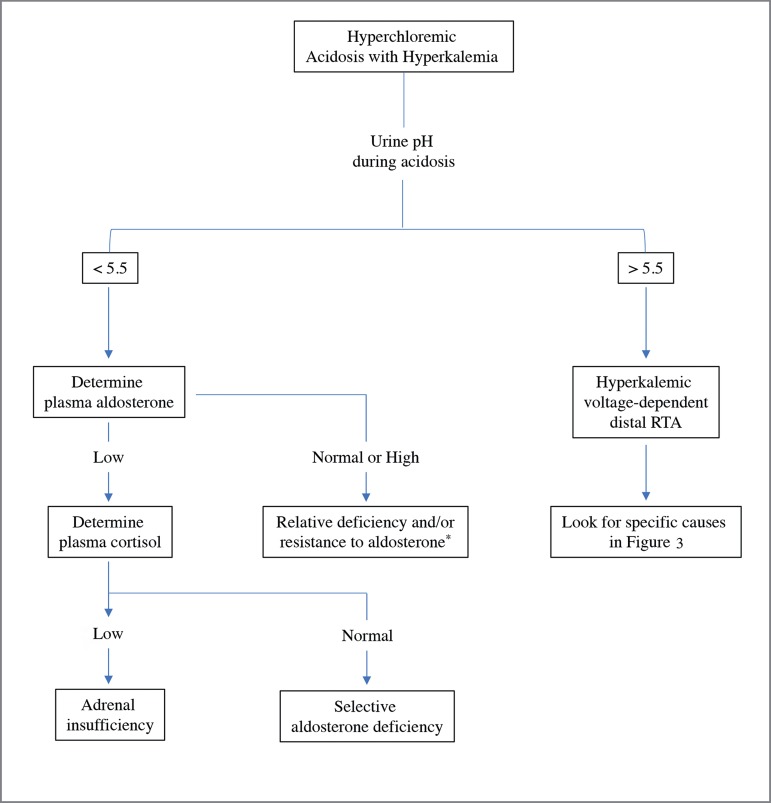
Clinical approach to the diagnosis of hyperkalemic RTA based on urine pH. Adapted from reference 1. *Antagonism, reduction or mutation in mineralocorticoid receptor.

It is well known that hyperkalemia raises intracellular pH by exchange with protons, impairing enzymes involved in ammoniagenesis and thus can *per se* lead to acidosis, but it usually does not reduce urine pH below 5.5. However, when another factor besides hyperkalemia reduces ammonia production and excretion during acidosis, as observed in real or apparent hypoaldosteronism, urine pH is reduced below 5.5. Therefore, patients with aldosterone deficiency/resistance can lower urine pH "normally" during acidemia, and this capacity is extremely useful in distinguishing this syndrome from the so-called voltage-dependent hyperkalemic RTA (Figure 4)^1^^,^ 2 ^,^ 6.

Interestingly, in our case, the first urine collected presented pH of 5.0, suggesting the presence of aldosterone deficiency/resistance as shown in Figure 4. Plasma renin activity was increased while plasma aldosterone concentration was within the reference values (Table 1). When potassium is elevated, plasma aldosterone concentration should be at least three times higher^6^. Thus, an aldosterone of 13.8 ng/dL is a suboptimal hormonal response considering plasma potassium level of 9.25 mEq/L. Additionally, three months after the resolution of acidosis, when plasma potassium level was normal, plasma aldosterone was 39.1 ng/dL. These data support the existence of a relative and transient hypoaldosteronism.

PCM is the main systemic mycosis in Brazil caused by the dimorphic fungus *Paracoccidioides brasiliensis, which* predominantly involves the lungs but can disseminate to the mucous membranes, skin, lymph nodes, and adrenal glands. The frequency of adrenal involvement in PCM varies from 2.9% to 48% among the different clinical studies, but in necropsy reports, the adrenal invasion is as high as 85%-90% of the cases^7^. Severe hyperkalemia in a patient with previous diagnosis of PCM could be explained by Addison's disease. Although abdominal CT showed a poorly defined nodule in the left adrenal gland (3.1×1.9mm), there were no symptoms like hypotension, abdominal pain, hypoglycemia or hyperpigmentation of the skin. In addition, serum cortisol and ACTH levels were normal and aldosterone level became normal after withdrawal of the drugs. Thus, the hypothesis of hypoaldosteronism associated with PCM became unlikely.

Hyperkalemia and RTA are common complications that affect transplant recipients receiving immunosuppressive therapy with calcineurin inhibitors (CNIs) as cyclosporine and tacrolimus^8^^,^ 9. The mechanism of these adverse effects is multifactorial and related to CNIs serum levels. The most important one appears to be the inhibition of basolateral Na^+^/K^+^-ATPase at the CD^10^, which blocks sodium uptake by ENaC and causes the loss of lumen-negative potential difference, the so-called voltage-dependent mechanism, leading to reduced potassium and hydrogen secretion (Figure 2). NCC cotransporter stimulation, increased paracellular chloride reabsorption, and inhibition of ROMK channel in the distal nephron, via alteration of WNK kinases, can aggravate this effect^11^. It is suggested that CNIs-induced hyperkalemia is in part caused by cellular K^+^ leakage since erythrocyte membrane Na^+^/K^+^-ATPase activity is decreased and K secretory channels upregulated when these cells are incubated with CNIs^12^. Moreover, CNIs may reduce aldosterone production/secretion by direct action on the adrenal gland or associated hyporeninemia. Also, CNIs can create resistance to aldosterone's action by reducing mineralocorticoid receptor expression^13^^-^ 15. Finally, CNIs inhibit the polymerization of the hensin protein, which is responsible for converting bicarbonate-secreting b-intercalated cells into the acid secreting a-intercalated cells during metabolic acidosis^11^.

From the above, the marked increase in serum level of tacrolimus in this case (Table 1) can explain the hyperkalemic RTA by interfering with the voltage-dependent mechanism, hydrogen ion pump defect and by reduction of ammoniagenesis (Figure 3). The latter is caused by unappropriated level or resistance to aldosterone and by hyperkalemia itself, which together are responsible for the low urine pH at presentation. Delivery of Na^+^ did not seem to be the problem because there was an abundant excretion of this cation (U_Na_=117mmol/L), and the prompt response of hyperkalemia to bicarbonate infusion may point to a defect in generating a favorable electrochemical gradient in cortical CD as the cause of this syndrome. These findings are in line with a previous study in which TTKG significantly increased after bicarbonaturia induced by bicarbonate or acetazolamide administration, but did not normalize after mineralocorticoid administration, indicating tubular insensitivity to aldosterone^16^.

The reversible renal dysfunction related to acute CNIs nephrotoxicity occurs due to vasoconstriction of the afferent arterioles. It results from an increase in vasoconstrictor factors that include endothelin and thromboxane and activation of the renin-angiotensin system, as well as a reduction of vasodilator factors like prostacyclin, prostaglandin E2, and nitric oxide^10^. The process can explain the high urea/creatinine ratio suggestive of pre-renal injury and the high levels of renin as depicted in Table 1. Also, it demonstrates the different patterns of response in plasma renin activity with CNI, since hyporeninemic hypoaldosteronism is also found with these drugs. Thus, under certain conditions, dosage, and duration, the renin profile can change^17^. Elevated renin strengthens the hypothesis of a direct impairment of aldosterone production/secretion by the high level of tacrolimus. Furthermore, it is important to emphasize that RTA syndromes are characterized by a relatively normal GFR, and the degree of renal dysfunction found in the present case cannot be imputed as a causal factor for hyperkalemia.

Hypomagnesemia is an often neglected complication of CNIs in the post-transplantation period. These drugs induce renal loss of magnesium by reducing the expression of paracellin-1(claudin-16) in thick ascending limb cells and TRPM6 transporter in the distal convoluted tubule^10^^,^ 18. Interestingly, in clinical practice, the hypomagnesemia usually runs in parallel to hypokalemia since magnesium deficiency releases the magnesium-mediated inhibition of ROMK channels and increases potassium secretion^19^. However, the apparent paradox of concomitant hyperkalemia and hypomagnesemia can be detected in renal toxicity by CNIs. Another relevant fact is that ENaC and aldosterone blockers prevent renal Mg wasting by increasing membrane negative potential in distal nephrons and hypoaldosteronism tends to occur with hypermagnesemia^20^. Thus, the presence of hypomagnesemia associated to high FEMg (>4%) on admission was a key finding that indicated tacrolimus as the possible cause of hyperkalemia/hypoaldosteronism rather than the supposed adrenal insufficiency by PCM. Furthermore, hypomagnesemia may have contributed to acute nephrotoxicity of CNIs by aggravating renal vasoconstriction^10^^,^ 21.

Beta blockers have been described as a potential cause of type 4 acidosis, mediated by hyporeninemic hypoaldosteronism^22^. However, the high levels of renin in this case, eliminate the possibility of propranolol involvement as a causative factor.

Trimethoprim is a bacteriostatic antibiotic that has been related to the induction of hyperkalemia through the competitive inhibition of ENaC transporter, identically to the potassium-sparing diuretic amiloride. In addition, this drug also decreases Na^+^/K^+^-ATPase activity in the cortical CD^23^. Thus, trimethoprim limits the formation of a voltage gradient in the CD necessary to transepithelial excretion of potassium and hydrogen similar to tacrolimus. A previous case report also speculated that trimethoprim might have a direct effect on the adrenal axis, possibly inhibiting aldosterone synthesis/release, as the level of aldosterone was inappropriate for the hyperkalemia condition^24^. Thus, trimethoprim might play an adjuvant role in the induction of hyperkalemia in this case.

In summary, drug-nephrotoxicity and diseases such as diabetes and other conditions associated with underproduction of renin or aldosterone are the main causes of hyperkalemic RTA in clinical practice. It should be pointed out that urine pH is a cornerstone to the differential diagnosis of this disorder, suggesting aldosterone deficit/resistance as a causal factor when < 5.5. Clinicians must remain alerted to severe hyperkalemia, acidosis, and hypomagnesemia that might develop in patients undergoing therapy with CNIs. Besides, we emphasize that the CNIs combination with other drugs such as trimethoprim can aggravate hyperkalemia dangerously.
